# Case report: a fatal case of *Aspergillus felis* infection in an immunocompetent host

**DOI:** 10.1099/acmi.0.000453

**Published:** 2022-11-21

**Authors:** Jill Parkes-Smith, Michelle J. Bauer, Haakon Bergh, Anthony Eidan, Brian M. Forde, John Hilton, Timothy J. Kidd, Campbell Schmidt, Adam G. Stewart, Patrick N. A. Harris

**Affiliations:** ^1^​ Pathology Queensland, Brisbane, Queensland, Australia; ^2^​ University of Queensland, Brisbane, Queensland, Australia; ^3^​ Royal Brisbane and Women’s Hospital, Brisbane, Queensland, Australia; ^4^​ UQ Centre for Clinical Research, Brisbane, Queensland, Australia

**Keywords:** aspergillosis, *Aspergillus felis*, cryptic *Aspergillus *species

## Abstract

We report a fatal case of *Aspergillus felis *invasive rhinosinusitis with secondary cerebral abscesses in an immunocompetent host despite aggressive surgical debridement and combination antifungals. Whilst this organism is known to cause fatalities in cats, only a few cases in humans have been documented, all of which had significant immunosuppression. This is the first human death due to *A. felis* described in an immunocompetent host.

## Introduction

*Aspergillus felis* is a member of the *Aspergillus viridinutans* complex and is a rare cause of invasive aspergillosis in cats, dogs and humans. It is phenotypically indistinguishable from other species within this complex and as such can be misidentified [1]. With advances in molecular identification techniques and whole-genome sequencing (WGS), more cryptic *Aspergillus* species are identifiable. Few clinical cases of *A. felis* in humans have been described, which could be a result of cases being misidentified as a different species within the complex [1, 2]. The previously described cases in humans have been in the immunocompromised host and predominantly involving the sinopulmonary tract [3–5]. In cats and dogs, *A. felis* is more commonly associated with fungal rhinosinusitis and can cause lysis of the orbital lamina with localized invasion into the orbital cavity, resulting in exophthalmos [6]. Identification of this species has clinical significance, as *A. felis* often expresses higher levels of resistance to commonly used antifungals, including the azoles, and can be refractory to aggressive antifungal therapy and surgical debridement [1, 2, 6].

## Case report

An 18-year-old male with an unremarkable medical history presented to the emergency department with cranial neuropathies and a headache. He had a 5-month history of mild frontal headaches, self-managed with intranasal xylometazoline hydrochloride. He was a non-smoker, apprentice electrician, with a healthy pet dog. There was no history of recurrent childhood infection or family history of immunodeficiency.

**Fig. 1.**
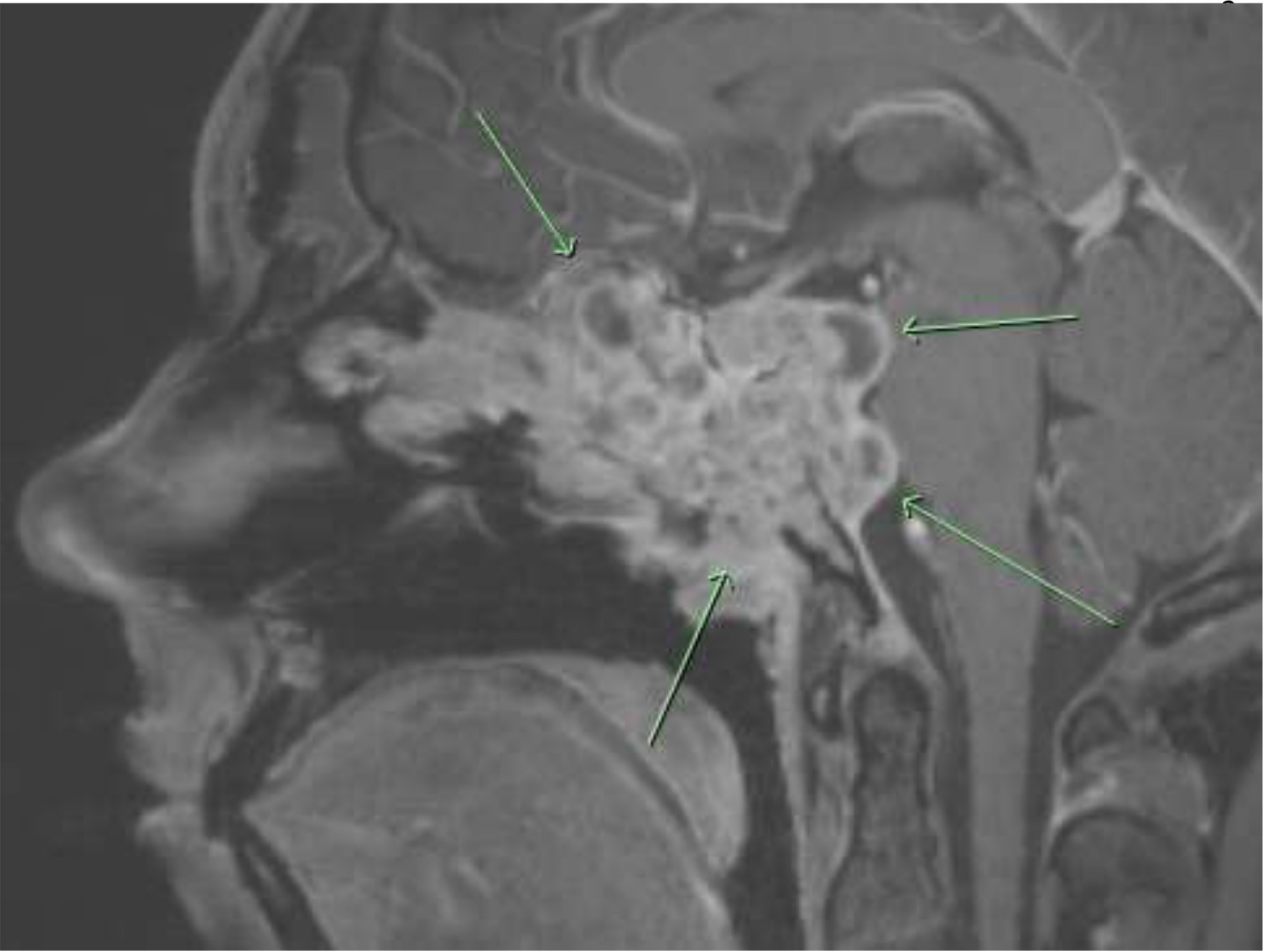
Invasive aspergillosis due to *Aspergillus felis* involving the base of skull.

On examination he had a right hypoglossal and left oculomotor nerve paresis with inflammatory polyps on nasoendoscopy. Magnetic resonance imaging (MRI) of his cranium demonstrated a large expansile mass centred on the clivus, expanding into the posterior fossa, pituitary fossa and anteriorly towards the nasopharynx and paranasal sinuses with significant erosion of the anterior skull base (Fig. 1).

Biopsy of right sinonasal tissue showed fungal elements with septate hyphae on periodic acid–Schiff staining. This was complicated by intra-operative haemorrhage from disruption of a suspected mycotic pseudoaneurysm of the right internal carotid artery. Intravenous posaconazole 300 mg twice daily and liposomal amphotericin B 10 mg/kg daily was commenced. The frequency of posaconazole was increased to three times a day after 1 week of therapy with trough plasma levels of 2 mg/L achieved. The patient had a mild neutrophilia (9.54×10^9^ /L) with a normal lymphocyte and eosinophil count. C-reactive protein was 2 mg/L. HIV testing was negative, serum protein electrophoresis was normal, IgG was 6.2 g /L and the other immunoglobulins isotypes were within the normal range. Cortisol was 16 nmol /L at 10 : 45 and ACTH <5 ng /L, but these results were in the context of recent steroid use. Oxidative burst studies yielded a normal response with unstimulated neutrophils of 1 % and stimulated neutrophils of 100 % (normal >90) and the stimulation index was 353 (normal >70). This assay was repeated and yielded a similar result. Lymphocyte subset studies revealed a CD3 of 0.24×10^9^/L (68 %), CD4^+^/CD3^+^ 0.12×10^9^/L (33 %), CD8+/CD3^+^ 0.11×10^9^/L (32 %), CD19 0.08×10^9^/L(23 %) and CD56 and CD16 0.02×10^9^/L (7 %).

Fungal growth occurred at 48 h on Sabouraud dextrose agar with microscopy demonstrating hyaline septate hyphae. White floccose colonies with central olive-green pigment developed after 1 week. A preliminary identification of *Aspergillus* species was reported. Clinical deterioration occurred over the course of 19 days, with repeat imaging demonstrating further intracranial abscess formation with secondary cerebral oedema and raised intracranial pressure. After wide consultation, life-sustaining therapy was withdrawn.

*A. felis* was identified using both internal transcribed spacer (ITS) region and WGS. Amplicon sequencing was undertaken using a 579 base pair fragment of the ITS using the primer pairs P-ITS-1 and P-ITS-4 [7]. Analysis using blastnr (http://www.ncbi.nlm.nih.gov/blast), MycoBank (https://www.mycobank.org) and MycologyLab (https://www.mycologylab.org/) yielded the identification of *A. felis* complex. For WGS fungal DNA was extracted using the Qiagen DNeasy UltraClean Microbial kit with the addition of lyticase to the PowerBead tube containing PowerBead and SL solution. This was preincubated at 70 °C for 10 min before bead beating. Library preparation was performed by the Illumina DNA Prep kit and sequencing by the Illumina MiniSeq platform. Kraken was used for taxonomic profiling with a custom database exclusively comprising *Aspergillus* genomes obtained from the National Center for Biotechnology Information (NCBI); this identified our isolate as *A. felis* [8]. Susceptibility testing was performed using broth microdilution [Clinical and Laboratory Standards Institute (CLSI)] [9]. The minimum inhibitory concentration (MIC) values for voriconazole, posaconazole and amphotericin B were 4, 0.5 and 2 mg/L, respectively, and are presented in Table 1.

**Table 1.** Antifungal susceptibility of *aspergillus felis* isolate using broth microdilution

Antibiotic	Minimum inhibitory concentration (mg l^−1^)
5-flucystosine	>64
Amphoteric B	2
Anidulafungin	0.03
Fluconazole	>256
Isavuconazole	4
Itraconazole	1
Micafungin	0.015
Posaconazole	0.5
Voriconazole	4

## Discussion

The *Aspergillus viridinutans* complex exists within *Aspergillus* section Fumigati. This includes six pathogenic species: *A. felis*, *A. udagawae*, *A. pseudofelis*, *A. parafelis*, *A. pseudoviridinutas* and *A. wyonmingesis* [10]. Species are morphologically indistinguishable and molecular techniques are required to identify them [1]. This organism was first identified in 2013, after being isolated from cats with fungal rhinosinusitis [1].

*A. felis,* has suede-like to floccose growth with grey to green patches of conidia [1]. The process to conidiation can be slow or sparse, which can lead to difficulty in identifying the organism in a timely manner, as occurred in this case. It is phenotypically indistinguishable from other species within this complex, although it may be differentiated from *A. viridinutans* by its growth at 45 °C and from *A. fumigatus* by its inability to grow at 50 °C [1]*. A. felis* conidial heads are uniseriate and columnar.

A previous study of 20 human and animal isolates of *A. felis* examined the use of ITS-1, ITS-2 and the 5.8S rDNA gene and parts of the β-tubulin (*benA*) and calmodulin (*calM*) gene [1]. ITS region sequence analysis for the identification of *A. felis* is a reliable method [1].The species within this complex are morphologically indistinguishable and as such molecular techniques are required to identify them to the species level Appropriately identifying these cryptic species in a timely manner is critical, as they typically have higher MICs for triazole antifungals, which are often the first-line therapy, and susceptibility testing is usually significantly delayed [1, 10, 11].

Invasive *A. felis* infection has mainly been described in dogs and cats, with only a few documented cases in humans, all of whom have been immunosuppressed. In cats, cases typically present with unilateral exophthalmos secondary to retrobulbar fungal granuloma or other manifestations of sinusitis, and in contrast to dogs and humans, it usually occurs in immunocompetent cats [1, 10]. Few cases of invasive disease in humans have been described in the literature. One case involved an immunosuppressed 56-year-old male with rheumatoid arthritis and diabetes mellitus, who developed chronic invasive pulmonary aspergillosis and died after an 18-month period of illness with the condition [12]. In this case the organism was initially identified as *A. viridinutans*, only to be later reidentified as *A. felis* [1]. Similarly, other case reports that have identified other cryptic *Aspergillus* species have been subsequently reidentified with the evolution of molecular methods to have been *A. felis* [1, 2, 13]. Two other cases of successfully treated invasive pulmonary aspergillosis with *A. felis* have been described in case studies, one involving a 42-year-old male with chronic granulomatous disease and the other a 41-year-old female with myelodysplastic syndrome who underwent autologous peripheral blood stem cell transplantation [3, 4]. A case of cranial aspergillosis with *A. felis* was reported in a 66-year-old male with chronic lymphocytic leukaemia on ibrutinib and was successfully managed with voriconazole and surgery followed by maintenance with posaconazole [5]. Another case involved a 40-year-old male with osteomyelitis of the lower limb, but clinical details are scarce [1]. As culture-based phenotypic identification can lead to misidentification, it is certainly arguable that other clinically significant cases of invasive aspergillosis caused by *A. felis* have been misidentified as *A. fumigatus*.

There is a paucity of information regarding the role of *A. felis* as a human pathogen and its propensity to cause invasive disease. Previous studies have identified that 7.5–10 % of all *Aspergillus* clinical isolates are cryptic species [14, 15]. As such, it is difficult to establish a true estimate of the local and global impact of this organism. As molecular and genomic methods of fungal identification become more incorporated into clinical laboratories, surveillance of this potential emerging pathogen will be important.
